# Use of Blockchain Technology for Electronic Prescriptions

**DOI:** 10.30953/bhty.v4.183

**Published:** 2021-10-22

**Authors:** Ryan W. Seaberg, Tyler R. Seaberg, David C. Seaberg

**Affiliations:** 1 Eirsystem Inc., Chattanooga, TN, USA; 2 Department of Emergency Medicine, Northeast Ohio Medical University, Akron, OH, USA

**Keywords:** blockchain, electronic prescribing, distributed ledger, electronic health records

## Abstract

**Objective:**

Distributed ledger technology can be used as a transparent, shareable ledger, that can record transactions between two parties efficiently and in a more secure, verifiable, and permanent way than the current electronic prescribing systems. We studied the use of a distributed ledger electronic prescribing programme, Prescription Abuse Greatly Reduced (PAGR) Prescriptions, to examine the effect of blockchain on provider prescribing efficiency at three family medicine clinics.

**Design:**

The PAGR was installed side-by-side to the electronic health record at three family medicine practice clinics in middle Tennessee. A prospective, convenience sample of patients at all three clinics was used for analysis. Trained observers were used in each clinic to document the side-by-side use of current prescribing practice versus the use of the PAGR electronic prescribing system by the individual providers.

The primary outcome was total time to write the prescription. Secondary metrics included compliance with checking the state’s Physician Drug Monitoring Program (PDMP.) , accuracy of medicine reconciliation, use of patient’s eligibility on insurance, prescription benefits, and change in prescription caused by benefits analysis or drug-interactions. Provider satisfaction was measure on a 4-point Likert scale.

Data were analysed using two-tailed, paired Student T-tests with alpha set at 0.05. A sample size of 107 patients was calculated to have a power of 80% to detect a 50% change in the prescription writing time.

**Results:**

The primary outcome of total prescription writing time was 171 ± 41 sec for current prescribing practice versus 63 ± 15 sec for the PAGR system (p = 0.0006). All providers were extremely satisfied with the use of the PAGR programme.

**Conclusion:**

Use of the PAGR electronic prescription programme significantly saved a mean of 1 min 48 sec per written prescription at the three Family Medicine Clinics. The PAGR also provided accurate medicine reconciliation and complete PDMP checks for controlled substance prescriptions. The patient real-time benefits check and drug-drug and allergy-drug reviews resulted in the provider changing the prescription 28% of the time, enhancing safety and out-of-pocket patient expenses. Future enhancements include expanding the insurance benefits analysis and developing provider notifications when patients are non-compliant with filling their prescriptions.

Ablockchain is a distributed database system that keeps track of records. As records are added to the blockchain, they are ordered in blocks, and each block contains a unique identifier and timestamp. These blocks are stored on each node of the network and are encrypted so that it is not possible to make changes later without changing all of the following nodes (1–3). Therefore, a blockchain can serve as an open, distributed ledger, that can record transactions between two parties efficiently and in a more secure, verifiable, and permanent way than the current electronic prescribing systems (4, 5).

Many clinicians feel that using electronic medical records and electronic prescription products detracts from clinical effectiveness (6). Many of the products are not designed to provide efficient workflow. Blockchain has the advantage of using distributive ledger technology to provide relational databases centred around the patient and more easily accessible by the clinician. We studied the use of a blockchain electronic prescribing programme to examine the effect on prescribing practices at three family medicine clinics. The blockchain programme, Prescription Abuse Greatly Reduced (PAGR) Prescriptions by EirSystem, Inc., was developed to use blockchain to write electronic prescriptions and to automate safety features such as checking the state’s Physician Drug Monitoring Program (PDMP) for controlled substances, provide real-time medicine reconciliation and insurance benefit analysis, and check drug-drug and drug-allergies interactions.

The PAGR software would ideally be acting as a single repository for all the necessary information to write an electronic prescription as shown in Fig. 1. Distributed ledger technology can bring in patient data from hospital, pharmacy and clinic sources, and combine it with state and federal databases to provide a more efficient workflow for the clinician. Current legacy systems often require the clinician to open multiple programmes or databases to examine this data, providing a disruption to their operation (7). By making this information accessible through one query search and not multiple, the user will identify better-prescribing trends and habits for their patients. Putting this information on a secure network provided by the distributed ledger, will ensure that access is only given to the appropriate credentialed end user, and that each transaction is providing all the necessary information to pass state and federal audits.

**Fig. 1:**
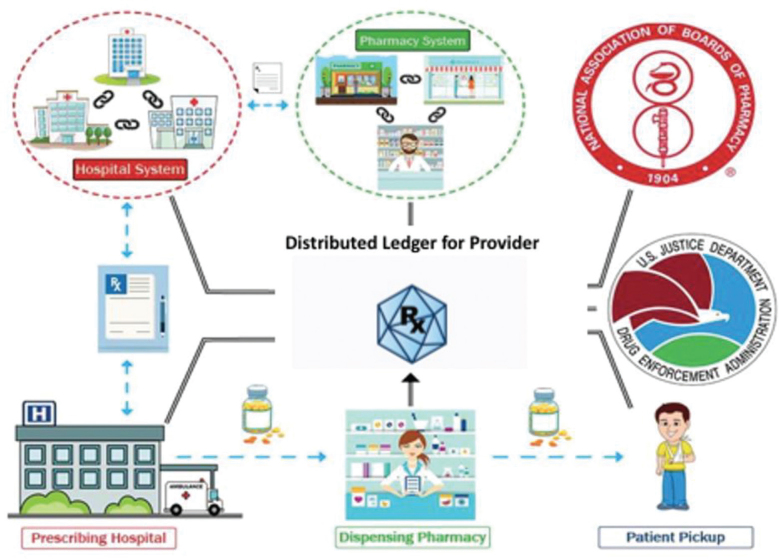
How EirSystems’ blockchain interacts with the current US healthcare system.

Our hypothesis was that the use of a blockchain electronic prescribing product, PAGR, would improve the functionality for the prescriber while maintaining data integrity and accuracy of medicine reconciliation.

## Methods

The PAGR programme was installed as a pilot programme at three different clinics in the Murfreesboro and Shelbyville, Tennessee area. These clinics were a combination of rural and semi-rural practices that saw a wide range of patient demographics. The clinics were staffed by two nurse practitioners and one physician assistant under the supervision of a single, board-certified family physician. A convenience sample of patients at all three clinics was used for the analysis of the data collected.

The PAGR was installed at all three clinics to run side-by-side with the existing electronic health record (EHR) to compare the PAGR prescribing platform’s performance against the legacy EHR prescribing capabilities. All three clinics utilised Practice Fusion as their legacy EHR system and prescribed drugs using their built-in electronic prescribing portal. Two Health Insurance Portability and Accountability Act (HIPAA)-certified observers were placed in each clinic to record data according to *a priori* established metrics. Data were collected prospectively by comparing the performance of both electronic prescribing platforms side by side for the same prescription then recording the differences in the metrics measured. The clinician would prescribe using the PAGR platform first, and then prescribe using their EHR platform. This was done to ensure consistency in protocol and to minimise any learning effect bias. The study was performed over 3 months, from December 2021 through February 2021.

The primary study outcome was the total time for prescribing a drug. A trained observer used a digital timer to record the time to prescribe on PAGR’s platform compared to the same time to prescribe on the Practice Fusion platform.

Secondary outcome measures included use of the state PDMP programme score, prescribing controlled or non-controlled substances, viewing of the eligibility and formulary checks, reviewing of therapeutic alternatives, use of the allergy-to-drug and drug-to-drug interactions, reviewing the patient’s prescription benefits based on the eligibility, and whether the formulary check influenced the prescription written. Additional metrics collected included the total number of active and inactive prescriptions, and the number of duplicate prescriptions. Accuracy of medicine reconciliation was done between the EHR and PAGR. Prescriber satisfaction was recorded using a 4-point Likert scale.

Data were analysed using two-tailed, paired Student T-tests with alpha set at 0.05 using the Statistical Package for the Social Sciences (SPSS) software. A sample size of 107 patients was calculated to have a power of 80% to detect a 50% change in prescription writing time. We felt that a 50% change in prescription writing time would be significant for the provider. A paired t-test is used to compare two populations means, where you have two samples in which observations in one sample can be paired with observations in a second sample, such as before-and-after observations on the same subject.

## Results

A total of 126 patients, ages 7–82 years old, were enrolled, with 107 prescriptions written over the 3-month study period. The primary outcome of total prescription writing time was 171 ± 41 sec for current prescribing practice versus 63 ± 15 sec for the PAGR system (*p* = 0.0006).

Using the PAGR system, medicine reconciliation was done on all patients with a mean of 11 ± 8 active and 88 ± 72 inactive prescriptions per patients. Accuracy of medicine reconciliation between PAGR and EHR was 96% (95% CI: 70–99.0%). Using the PAGR programme, prescription eligibility checks occurred in 81% of patients and benefits analysis was available in 22.6% of prescriptions, none of which occurred using the site’s EHR. Drug-to-drug or allergy-to-drug interactions occurred in 58% of patients (95% CI: 48.8–66.6%). Using PAGR, a therapeutic alternative was offered in 41% of prescriptions and prescribers changed the prescription 30 times (28.0%, 95% CI: 19.8–37.69%) based on the drug or allergy interactions or from benefit analysis. Again, this did not occur using the site’s EHR. A controlled drug prescription was written 72 time and the PDMP or NarxCare database was checked in all cases using the PAGR. On the site’s EHR, the prescriber had to access a separate platform to view the PDMP report which resulted in no compliance. All providers were extremely satisfied with the use of the PAGR programme (Likert score of 4.0).

## Discussion

Blockchain is a system that records various transactions on an immutable ledger across a trustless peer-to-peer network. Every transaction enacted stores specific data on a ‘block’ that cannot be altered once sent across the network, creating a ledger that gives a complete record of all transactions. Every transaction creates a new block added to the chain; the more transactions one has on the chain, the more secure it becomes. Once a transaction has been enacted and the appropriate data has been recorded, a timestamp is then placed on the block. This transaction time can then be tracked on the ledger (1, 3, 5). This allows the ease of recording data more securely and provides the tools to track any transaction enacted on a network. The blockchain will enable users to see the exact time someone performed a transaction and any other data that is recorded, which cannot be altered. This process is inherently more secure than traditional centralised data systems. The blocks are stored on each node of the network, and are encrypted so that it is not possible to make changes later without changing all the following nodes (1, 5). The peer-to-peer network that gives blockchain its myriad benefits is governed by smart contracts. These smart contracts are instructions given to the network of peer-to-peer nodes that automate transactions across a vast network with different rules and regulations, but all governed by similar logic. These smart contracts can be tailored for individual users based on their current needs. Therefore, a blockchain can serve as an open, distributed ledger, that can record transactions between two parties efficiently and in a more secure, verifiable, and permanent way than the current electronic prescribing systems (4, 5).

The use of blockchain technology in medicine is increasing exponentially (8). Adoption of block chain technology could bring both potential costs and savings. Unfortunately, healthcare today suffers from fragmented datasets, delayed communications, and disparate workflow issues caused by lack of interoperability (5, 9). Blockchain allows data across multiple independent systems to be accessed simultaneously and immediately by those with sufficient permissions. This interfacing of different systems reduces medical and financial errors, and reduces administrative delays (9, 10). The use of smart contracts allows patients’ consent preferences to be executed immediately, further reducing administrative costs (9, 11). Additionally, blockchain supports high-throughput data analysis as well as machine learning artificial intelligence (AI) strategies (12). Blockchain represents an innovative vehicle to manage medical records, ensuring interoperability, but without compromising security. It also protects patient privacy, allowing patients to choose who can view their data. Investments into this technology would be outweighed by returns as the interfacing of systems leads to increased collaboration between patients and healthcare providers and improved healthcare outcomes.

Through contracted third-party software such as Surescripts and Appriss as well as pharmacy benefits managers like CVS Caremark and BlueCross BlueShield, it was established that the PAGR programme interacts with various databases that are critical for processing the necessary data to complete an electronic prescription. These connections to third party vendors are engineered through a Nexus application programming interface (API) that facilitates the data following the National Council for Prescription Drug Programs (NCPDP SCRIPT Standard version 2017071). Engineering the backend software, allows the ability to transact data amongst verified third party vendors because NCPDP SCRIPT Standard is the national standard that electronic prescription software’s must abide by. The PAGR programme has also received Electronic Healthcare Network Accreditation Commission-Electronic Prescribing of Controlled Substances (EHNAC-EPCS) certification, which details the required steps to verified medical personnel to transact personal health information (PHI) data. Building out the software to connect with other vendors via Representational State Transfer Application Programming Interface (REST APIs) allow the software to have more flexibility as an integrator than it would to build out the connections directly to the blockchain. As a result of the PAGR’s Nexus REST API infrastructure, the user is interacting with our user interface (UI), that is calling on the REST APIs to present all of the necessary information to write and finalise a prescription. What is distinctive about the PAGR platform is that before the operating system sends off a prescription to the pharmacy, it sends a copy of the prescription data to the PAGR’s blockchain. The blockchain then runs its programmed Chaincode or ‘smart contracts’ to verify that all the data is valid. This means that our blockchain ensures that the unique credential tied to the prescriber is valid to write a prescription, there are no serious interactions or complications with the patient taking the medicine, that the prescriber has checked all the necessary databases before finalising, and that the drug is not being overprescribed.

These features allow the PAGR platform to bring in better and more concise data around the patient. By building a UI that was designed by doctors, the programme can take this data and display it in ways that will impact the workflow of the user (13). Another benefit that PAGR brought to its users was the ability to confirm and use correct data (such as insurance and benefits) in the prescribing process, which saved prescribers time and helped influence the decision of the prescriber. The PAGR platform was also able to evolve the workflow of the prescriber because every time the prescriber wrote a prescription and it was stored on the blockchain, the prescriber could utilise that data again for the patient knowing that it is correct. Additionally, the PAGR programme contains all the necessary certifications to handle PHI, both domestically and internationally. The integrated cloud services are Federal Risk and Authorization Management Program (FedRAMP) certified, and every transaction is performed through end-to-end encrypted portals using Transport Layer Security (TLS) layer 2 to transact the data. These credentials allow transactions at a federal, state, and international level.

The use of blockchain technology for electronic prescribing is in its infancy (14). System ease of use is often measured in time and functionality in accessing the needed information to treat the patient. Use of the PAGR electronic prescription programme significantly saved a mean of 1 min 48 sec per written prescription during our study at the three Family Medicine Clinics. The PAGR also provided accurate medicine reconciliation, provided complete PDMP checks for controlled substance prescriptions and their benefits, and drug-drug and allergy-drug checks resulted in the provider changing the prescription 28% of the time, enhancing safety and out-of-pocket patient expenses. Use of blockchain technology in electronic prescriptions brings disparate databases around the patient to provide better useability for the practitioner, reducing time spent writing prescriptions, and enhances patient safety. This benefits both the user and the healthcare system. Future enhancements include expanding the insurance benefits analysis and developing provider notifications when patients are non-compliant with filling their prescriptions. We will also want to add over the counter drugs to our medicine reconciliation functionality, so we provide a complete detailed list of the patient’s medications. This will allow our software to have better information to run the drug-drug and allergy-drug checks.

## Limitations

The use of only three small family practice sites limits the external validity of the study. Since this was a newly developed programme, we needed a controlled but limited practice setting to test the proof of concept for the use of the blockchain in electronic prescribing. There are also concerns about the effect of learner bias in measuring prescription writing time. This was minimised by our study design that made the provider use the PAGR programme first, and then prescribing using their EHR platform. If anything, this biased against the PAGR system in prescription timing, perhaps underestimating the significant time difference found between the two systems. Lastly, there are no standard metrics for comparing electronic prescription programmes. We used time for prescription, ease of use and completeness of the medicine reconciliation as a proxy for programme effectiveness. This study was designed to measure effectiveness from the prescribers’ viewpoint. Our study tried to show the workflow enhancements with a description of the improved security and data management of blockchain technology for electronic prescriptions.

## References

[1] Epalm. Blockchain in Healthcare [Internet]. HIMSS; 2021 [cited 2021 Jan 23]. Available from: https://www.himss.org/resources/blockchain-healthcare

[2] Zhang P, Schmidt DC, White J, Lenz G. Blockchain Technology use cases in healthcare [Internet]. Advances in Computers. Elsevier; 2018 [cited 2021 Feb 2]. Available from: https://www.sciencedirect.com/science/article/pii/S0065245818300196

[3] Engelhardt MA. Hitching healthcare to the chaIn: An introduction to Blockchain Technology in the healthcare sector [Internet]. Technology Innovation Management Review. TIM Review; 2017 [cited 2021 Jun 20]. Available from: https://timreview.ca/article/1111

[4] Parker M, St. Clair J. Distributed Ledger Technology and Blockchain Solutions for interoperability [Internet]. HIMSS; 2020 [cited 2021 Jan 21]. Available from: https://www.himss.org/resources/distributed-ledger-technology-and-blockchain-solutions-interoperability

[5] Vazirani AA, O’Donoghue O, Brindley D, Meinert E. Blockchain vehicles for efficient Medical Record management. NPJ Digit Med. 2020;3:1. doi: 10.1038/s41746-019-0211-0 31934645 PMC6944683

[6] The Harris Poll. How doctors feel about electronic health records [Internet]. https://med.stanford.edu › EHR-Poll-Presentation. Stanford Health; [cited 2021 Jun 14]. Available from: https://med.stanford.edu › EHR-Poll-Presentation

[7] Porterfield A, Engelbert K, Coustasse A. Electronic prescribing: Improving the efficiency and accuracy of prescribing in the ambulatory care setting [Internet]. Perspectives in health information management. American Health Information Management Association; 2014 [cited 2021 Jun 20]. Available from: https://www.ncbi.nlm.nih.gov/pmc/articles/PMC3995494/PMC3995494 24808808

[8] Gordon W, Wright A, Landman A. Blockchain in health care: Decoding the hype [Internet]. NEJM Catalyst; 2017 [cited 2021 Jun 20]. Available from: https://catalyst.nejm.org/doi/full/10.1056/CAT.17.0523

[9] Vazirani AA, O’Donoghue O, Brindley D, Meinert E. Implementing blockchains for efficient health care: Systematic review. J Med Internet Res. 2019;21(2):e12439. doi: 10.2196/12439 30747714 PMC6390185

[10] Mamoshina P, Ojomoko L, Yanovich Y, et al. Converging blockchain and next-generation artificial intelligence technologies to decentralize and accelerate biomedical research and healthcare. Oncotarget. 2017;9(5):5665–90. doi: 10.18632/oncotarget.22345 29464026 PMC5814166

[11] Sanka AI, Irfan M, Huang I, Cheung RCC. A survey of breakthrough in blockchain technology: Adoptions, applications, challenges and future research. Comput Commun. 2021;169:179–201. doi: 10.1016/j.comcom.2020.12.028

[12] Benjamens S, Dhunnoo P, Meskó B. The state of artificial intelligence-based FDA-approved medical devices and algorithms: An online database. NPJ Digit Med. 2020;3(1):118. doi: 10.1038/s41746-020-00324-0 32984550 PMC7486909

[13] Javed I, Alharbi F, Bellaj B, Margaria T, Crespi N, Qureshi K. Health-ID: A Blockchain-based decentralized identity management for remote healthcare. Healthcare. 2021;9(6):712. doi: 10.3390/healthcare9060712 34200778 PMC8230390

[14] Seitz J. Blockchain technology in e-health: The case of electronic … [Internet]. University of Novi Sad; [cited 2021 Mar 2]. Available from: https://www.iim.ftn.uns.ac.rs/is17/papers/28.pdf

